# Influence of the Variability of Calcareous Fly Ash Properties on Rheological Properties of Fresh Mortar with Its Addition

**DOI:** 10.3390/ma12121942

**Published:** 2019-06-17

**Authors:** Artur Nowoświat, Jacek Gołaszewski

**Affiliations:** Faculty of Civil Engineering, Silesian University of Technology, 44-100 Gliwice, Poland; jacek.golaszewski@polsl.pl

**Keywords:** fly ashes, rheological properties, mortar workability, concrete, concrete additives

## Abstract

One of the main by-products of brown coal burning is calcareous fly ash (CFA). Apart from other applications, it is used as a main component of cement, or as an active mineral additive to concrete. The present study involves the impact of the raw and processed calcareous fly ash (CFA) on the changes of rheological properties of mortars. The said impact was determined by using the coefficient of variation (CV) given in percentage. CFA samples collected from various sources were subjected to testing. The samples were collected from two electrofilters of blocks with boilers of different combustion parameters and from the retention tanks of the CFA sales department (CFA T) in which CFAs from various boilers are mixed. It has been demonstrated that the degree of the impact of CFA addition on the rheological properties of mortars depends on the source of this addition and on bulk density. The present work demonstrates the negative impact of raw CFA on mortar workability, and hence it is questionable to support its use in concrete technology.

## 1. Introduction

In Poland calcareous fly ashes (CFAs) are mainly by-products of the combustion of brown coal in boiler furnaces of power plants. CFAs are precipitated on electrostatic precipitators. The resources of CFA in Poland are large; its annual production exceeds 4.5 million tons. Consequently, there is an important problem involving its utilization, because such significant amounts pose threat to the natural environmental. One of the possible ways to utilize CFA is to use it as a main constituent of common cements, or an active mineral additive to concrete. In the studies carried out to date [1], it has been demonstrated that with respect to CFA available in Poland, the most favourable properties in terms of its application in cement or concrete technology has the CFA from the Bełchatów power plant. That CFA meets the requirements of the standard EN 197-1 [2] on main constituents of common cement, and after the grinding process it can be also used as an active mineral additive to concrete [1,3,4,5]. As demonstrated in systematic and very extensive studies [5,6,7,8,9,10,11], the use of calcareous fly ash of up to 30% of cement, both as an additive to cement and as an additive to concrete, generally does not adversely affect the strength or durability properties of concrete. It has also been declared that CFA subjected to the grinding process can even have a beneficial effect on the selected properties of concrete, such as permeability, resistance for chloride corrosion or expansion caused by alkali-silica reaction ASR. Besides, the view on the demand for these cement additives was expressed in the article by Argiz et al. [12]. In this context, as the main ingredient for Portland cements, they used bottom ashes as an optimal mixture with fly ash. Previously, such research studies on bottom ash was conducted by Cheriaf et al. [13].

Unfortunately, the use of CFA also poses considerable problems. In a raw state, CFA is characterized by very high water demand [4]. It can be reduced by the grinding process. Yet, even then it remains higher than the water demand of cement [1,5]. High water demand of CFA makes it difficult to obtain fresh concrete with the required and stable workability in the long term [5,11,12,13,14,15,16,17,18]. This problem can be largely resolved by using effective plasticizers and superplasticizers [5,19,20,21,22,23,24]. Another disadvantage, perceived as a serious difficulty, is the high variability of chemical composition, physical properties (such as fineness and water demand) as well as other properties important for practical reasons in terms of CFA application [1]. Although significant improvement has been observed in the past few years, it is still significantly higher than that found in the case of siliceous fly ashes (SFA) [1]. So far, however, the problem involving the impact of CFA composition variability and properties on the properties of fresh concrete has not been subjected to systematic research.

This paper presents an investigation on the influence of the variability in CFA properties on the variability of rheological properties of cement mortars [1,20]. Rheological properties of mortars were tested using rheometric techniques. A dozen or so CFA samples were being collected from different sources (14 electrostatic precipitators and 10 retention tanks) over three months. CFA was used as partial cement replacement in the amount of 20% in the raw form or processed by grinding.

## 2. Experimental

### 2.1. Research Plan and Scope

The objective of the undertaken research was to determine the impact of the variability of raw and processed CFA properties on the rheological properties of mortars and their variability. The analysis of CFA properties included its chemical composition, loss on ignition (LoI), content of CaO_free_, fineness determined as a residue on a 0.045 mm sieve along with water demand (both determined according to EN 450-1) and bulk density. The following CFA samples collected from different sources were examined: (1) from two electrostatic precipitators of power generation blocks with boilers characterized by different combustion parameters (CFA B1 and B2, CFA B2 from power generation blocks with the boiler with lower combustion temperature); and (2) from the retention tanks of the CFA sales department (CFA T) in which CFA from various boilers (not only B1 and B2) are mixed. The CFA samples from the electrostatic precipitators were collected once a week (10 samples from each precipitator were investigated) and from the retention tank once a month (4 samples). The CFA was used as a replacement for a part of the cement in an amount of 20% by weight.

Testing the rheology of concrete is a time consuming and expansive process. It is also difficult to control the influence of external factors on the measurement results and its variability. The studies on the rheology of mortars and concretes indicate that the results of rheological measurements obtained for mortars (although the effect of the coarse aggregates is significant) are suitable for the prediction of fresh concrete rheology, and that the measure of the rheological parameters of the mortars can be directly related to the equivalent parameters in concrete [25]. Therefore, we decided to evaluate the influence of CFA variability, using mortars.

The influence of CFA on the variability of rheological properties of mortars was defined using the coefficient of variation (CV), given in percentage. It was calculated as a quotient of the absolute measure of the variability of a given rheological parameter effected by the diversity caused by CFA properties (defined as a standard deviation σ) and the average value of this characteristic. It was assumed that if the CV for the rheological parameters of mortar effected by a given factor was higher than 10%, this factor influenced the variability of rheological properties. If the CV was higher than 15%, the influence of this factor was very strong.

According to [26] the average values of CV (CV_av_) for a series of measurements of rheological parameters performed under the same, strictly controlled conditions are 4.7% and 4.2% for the yield value (g) and the plastic viscosity (h), respectively (CV_g max_ = 6.5%, CV_h max_ = 8.1%). Therefore, if the CV for the series of rheological parameters of mortars with different CFA was higher than CV_av_ and lower than 10%, this proved a possible influence of the variability of CFA properties on the rheological properties of mortars. Yet, this influence was insignificant for practical reasons. If the CV was higher than 10%, it meant that the variability of the CFA was significant and it significantly influenced the control of rheological properties of fresh mortars and concretes, without denying its applicability. However, if the CV was higher than 15%, it should be accepted that the variability of CFA properties significantly hindered the use of CFA in concrete technology. As a reference point for assessing the influence of CFA variability, we can also consider the influence of the variability of both commercial cements on the rheological properties of mortars [26]. According to [26] when we use CEM I and CEM III cements from the same source, but from different production batches over two months, the CV for rheological parameters ranges from 7% to 19%.

### 2.2. Material Properties and Mortar Composition

The documentation of the results of CFA variability with respect to the mentioned above properties is presented in Table 1. The composition of ashes and the variability of their properties did not differ from those presented in [1], so their high variability was confirmed. The variability of the CFA properties, taking into account the source of their collection, was evidently lower, but still it should be considered as high. The lowest variability of the properties was demonstrated by CFA T, collected from the retention tank. For practical reasons, this is an important observation. This shows that mixing the CFA from different sources in the retention tank to some extent eliminated the significant differences in their properties that may appear at this time. Thus, it allowed a material with more stable properties to be obtained. ANOVA involving the influence of the source of CFA on its properties is shown in Table 2 and Table 3. The CFA used in the study and taken from different sources did not differ significantly in terms of the parameters of chemical composition and fineness. It is worth noting that CFA B2 differed significantly from others in terms of bulk density and the loss on ignition (LoI). This effect was probably related to lower combustion temperature in block B2. Lower combustion temperature contributes to higher content of unburned coal, and it also affects the morphology of CFA grains and their glass transition [1]. It should be noted that the loss on ignition (LoI) in CFA samples is low, usually much lower than 5%. Thus, the tested CFA corresponds to the A class according to EN 450-1 [21] (max 5%). Raw CFA is always characterized by fineness higher than 40%, which means that is does not meet the requirements of EN 450-1 for fineness (40% for N class and 13% for S class). Obviously, the failure to meet the quoted standard does not rule out the possibility of using fly ashes in the concrete other than the type II additive. It should be also emphasized that the research was of cognitive and exploratory nature, carried out in order to acquire new knowledge on the possibility of applying calcareous fly ash as a component of concrete or cement, both in the unprocessed and processed form. The effects involving the use of unprocessed CFA were viewed as a reference point for the effects involving the use of the processed ash. Since fly ash is a by-product of a coal combustion process, which is optimized for the acquisition of maximum energy output, it seems impossible to optimize this process also in terms of fly ash quality without damage to its main purpose.

Thus, for CFA the application of its processing is necessary, preferably by grinding [1,5]. The CFA was processed by milling in a ball mill. In the case of CFA B1 and B2 (taken from the electrostatic precipitators), the objective, after grinding, was to obtain CFA with the fineness of 5% ± 2%, and in the case of CFA T (taken from the retention tank) the fineness of 20% ± 3%. In the latter case, the target fineness was inspired by the results presented in References [4,11], indicating an insignificant effect of grinding degree of CFA to a fineness below 20% on CFA activity and mortars rheology, and at the same time aiming to reduce the costs of CFA processing. The raw CFA used in the study considerably increased water demand in the range from 116% to 126% for CFA B2 and from 106% to 114% for CFA B1 and CFA T. The processing resulted in the drop of CFA water demand; after grinding it was not higher than 106%, and the highest water demand was for CFA B2. In general, it can be considered as acceptable since the standard ASTM C618 allows for the maximum increase of water demand to 105% in the case of Portland cement CEM I. The beneficial effect of grinding should be related to the change in the morphology of CFA grains. In the raw state, CFA contains a high amount of large grains with a very developed surface and with a large number of open pores. Such grains physically bind a large amount of water. In the case of processing by grinding, large grains are broken down and destroyed, which results in the decrease of water demand of CFA [14].

The composition and properties of the cement used are shown in Table 4. The composition of mortars used in the research is presented in Table 5. The composition of the mortars was based on the composition of standard mortars according to EN 196-1, but with water-cement ratio w/c = 0.55 to obtain mortars of fluidity adequate for the measuring range of Viskomat NT. Standard sand was used, according to EN 196-1, to avoid the influence of variability of sand grading on the rheological parameters of mortars [26].

### 2.3. Testing Method of the Rheological Properties of Mortars

Rheological behaviour of mortar, as of fresh concrete, may be sufficiently described by the Bingham model according to the equation:

(1) $${\tau \;=\;\tau }_{o}+\;\eta_{\mathrm{pl}}\dot{\gamma }$$

where: τ (Pa) is the shear stress at shear rate $\dot{\gamma }$ (1/s) and τ_o_ (Pa) and η_pl_ (Pas) are the yield stress and plastic viscosity, respectively [27]. The yield stress τ_o_ determines the stress above which the material becomes a fluid. It is of particular importance for fresh concrete workability—its value determines the accurate performance of technological processes of concrete production and construction concreting. The plastic viscosity η_pl_ is a measure of how easily the material will flow, once the yield stress τ_o_ is overcome; the higher the plastic viscosity η_pl_ of the mixture is, the slower is its flow. The meaning of the plastic viscosity η_pl_ in concrete technology is negligible in the case of ordinary concrete, but it is of significance for high flow and self-compacting workability and stability of concretes. The principles of rheology and rheological measurements are presented in the existing literature i.e., References [27,28]. The Schleibinger rotational Viskomat NT rheometer was used. For this rheometer, the Bingham equation is used in the conventional form:

M = g + h N(2)

The measurement of rheological parameters of the mortar consists in the determination of the torque M (N·mm) on a stationary probe mounted concentrically in a cylindrical container with the sample rotating with different speeds N (1/s). On this basis, using the method of least squares, it is possible to determine an equation of the M–N curve, and thus the rheological parameters: yield value g (N·mm) and plastic viscosity h (N·mm·s) corresponding to the yield value τ_o_ and plastic viscosity η_pl_ respectively. After determining the measurement constants of the rheometer, we can, if necessary, represent the values g and h in physical units. According to [29], in the apparatus like the one used in this work, τ_o_ = 7.9 g and η_pl_ = 0.78 h.

The mixer and the mixing procedure of the mortars were compliant with EN 196-1; CFA was added together with cement. After the completion of the mixing process, the samples of mortars were transferred to the Viskomat NT rheometer, and the M–N curve determined for *n* changed from 120 to 20 rpm. The measurements were carried out after 5, 30, 60 and 90 min since the end of the mixing. Having completed each measurement, the mortars were stored in the mixer and remixed for 2 min before the next measurement. The mortars were prepared and the measurements were performed in an air-conditioned room at the temperature of 20 °C ± 1 °C. The temperature of the mortars during the measurements was maintained at 20 °C ± 1 °C using a thermostatic device. The measurements were performed by one operator, generally without repetitions, except for the reference mortar REF (mortar without CFA) for which three repetitions were performed and for the selected CFA mortars for which a control repetition measurement was performed.

## 3. Results and Discussion

**Figure 1:**
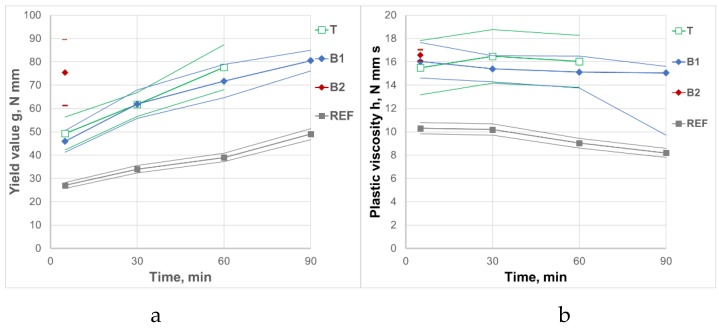
Influence of raw CFA on rheological properties of mortars and its variability. (**a**) Yield value g; (**b**) Plastic viscosity h.

**Figure 2:**
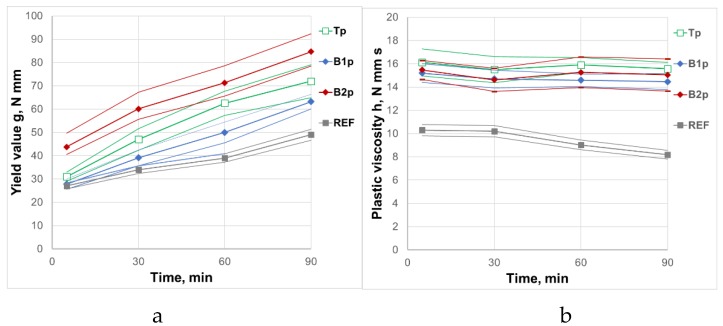
Influence of processed CFA on rheological properties of mortars and its variability. (**a**) Yield value g; (**b**) Plastic viscosityh.

Rheological parameters of REF mortar are presented in Table 6. The influence of raw and processed CFA variability on rheological parameters of mortars and its variability is presented in Table 7. ANOVA for the influence of the CFA source on the rheological parameters of mortars is presented in Table 8. The general influence of raw and processed CFA from different sources on the rheological parameters of mortars and its variability are shown in Figure 1 and Figure 2.

The research confirmed that the addition of raw CFA as a replacement of a part of the cement caused a significant increase of the yield value g and plastic viscosity h of the mortars, and in consequence it strongly and negatively influenced concrete workability. Moreover, raw CFA sped up the increase of the yield value g in time Δg, whereby only in a few cases the CFA mortars maintained plasticity, allowing the rheological measurement to be taken up to 60 min. The source of CFA had a significant impact on the effects of its addition. It is noteworthy that the source of CFA significantly affected the yield value g of the mortars, without affecting their plastic viscosity h. Rheological properties of the mortars were particularly negatively affected by CFA B2. This CFA was characterized by significantly higher water demand than CFA taken from other sources. In their case, as many as 60% of mortars showed no plasticity, and it was impossible to measure rheological properties for them even immediately after mixing. The coefficient of variation for the yield value g of raw CFA mortars after 5 min, without taking into account the source of CFA (all CFA), was 25%. This demonstrates a significant impact of the variability of raw CFA properties on the variability of rheological properties of the mortars, and moreover, it makes it very difficult to use the CFA in concrete technology. When using raw CFA from a specified source, the coefficient of variation for the yield value g of CFA mortars after 5 min decreased to the level of 8–14% (it should be noted that in the case of mortars with CFA B2, only 40% of measurements were possible). It is worth noting that the coefficient of variation for plastic viscosity h of raw CFA mortars was lower than 10%. It means that the variability of the composition and properties of raw CFA did not affect the plastic viscosity h of the mortars.

The research confirmed the beneficial effect of processing CFA by grinding. The presence of processed CFA still had a negative effect on the plasticity of mortars, but due to lower water demand of the processed CFA, this effect was clearly smaller than that for raw CFA. The addition of processed CFA brought about the increase of the yield value g and plastic viscosity h of the mortars. The increase of the yield value g was significantly lower when the raw CFA was added. By introducing 20% of raw or processed CFA, the yield value g increased, on average, by 92% and 29%, respectively. A clearly higher increase in the yield value g was due to the use of ground CFA B2p. After the grinding process, water demand of CFA B2p was still higher than that of ground CFA B1p and CFA Tp. Processing of CFA had an insignificant influence on the plastic viscosity h of the mortars. Processing made it possible to obtain mortars containing CFA whose acceptable workability was maintained for at least 90 min. However, the range of changes of the yield value g in time Δg of the mortars with ground CFA remained higher than that of the reference mortar REF without CFA. The highest increase of the yield value g in time Δg was observed for mortars with CFA B2p, and the lowest for mortars with CFA B1p, but the differences were not very significant. As in the case of raw CFA, the use of processed CFA had an insignificant effect on the changes in plastic viscosity h in time. The coefficient of variation for the yield value g of the mortars with processed CFA, without taking into account the source of CFA (all CFA), ranged from 23% to 15%, which was only slightly lower than in the case of raw CFA. The coefficient of plastic viscosity h of these mortars was lower than 10%. It means that as in the case of raw CFA, the variability of the composition and properties of raw CFA did not affect the plastic viscosity h of the mortars. However, if the source of CFA is taken into account, the coefficient of variation of the yield value g of the mortars decreased to the level below 10%, only in the case of CFA B2p, slightly exceeding it. This is particularly important in the case of CFA T, taken from retention tanks. It means that the use of CFA after processing by grinding enables the production of fresh concrete with stable, repeatable rheological properties. However, this requires a control of the CFA source.

**Figure 3:**
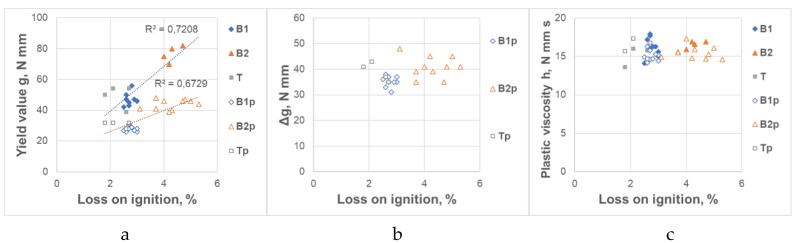
Influence of the loss on ignition of CFA on rheological properties of CFA mortars. (**a**) Yield value g; (**b**) Increase of the yield value g in time Δg, (**c**) Plastic viscosity h.

**Figure 4:**
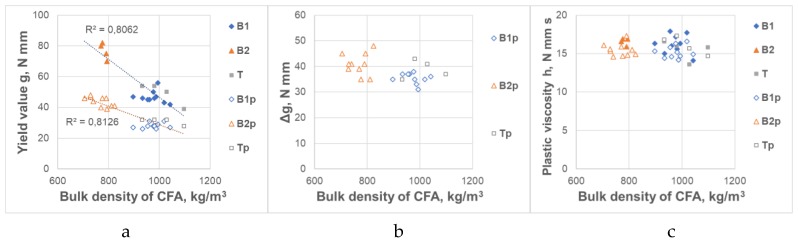
Influence of CFA bulk density on rheological properties of CFA mortars. (**a**) Yield value g; (**b**) Increase of the yield value g in time Δg, (**c**) Plastic viscosity h.

The influence of CFA on the rheological properties of mortars and their variability depends primarily on the CFA source, and so on the combustion parameters of the coal. The lower combustion temperature in the boiler, the higher the loss in ignition of CFA (higher the unburnt coal content) and lower CFA bulk density. As stated in Section 2.2, raw CFAs taken from various sources did not differ from each other in terms of chemical composition and fineness. However, as stated in Reference [5], the lower combustion temperature contributes to different morphology of CFA grains—they are more porous with a larger surface area, a higher content of unburnt coal and a higher content of amorphous phases. The nature of the impact involving the loss on ignition (LoI) and bulk density of CFA on rheological properties of mortars is shown in Figure 3 and Figure 4. As the loss on ignition (LoI) increased and the bulk density decreased, the yield value g and the changes of the yield value g in time Δg of the CFA mortars increased. These properties of CFA do not affect the plastic viscosity h of the CFA mortars.

**Figure 5:**
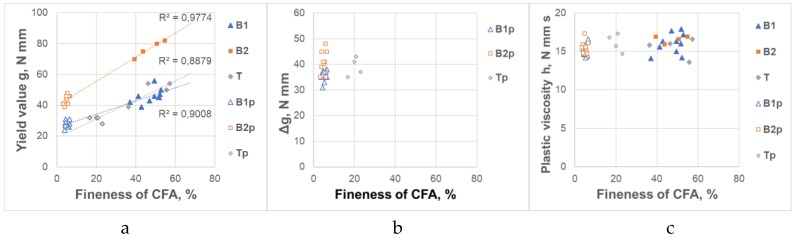
Influence of fineness of CFA on rheological properties of CFA mortars. (**a**) Yield value g; (**b**) Increase of the yield value g in time Δg, (**c**) Plastic viscosity h.

Another factor of a significant importance for rheological properties of CFA mortars and their variable rheology was the CFA fineness. The nature of the influence of CFA fineness on the rheological properties of mortars is presented in Figure 5. The processing effect on CFA mortar rheology is obvious, but it is clear that the addition of CFA with higher fineness, whether raw or ground, gave mortar with a lower yield value g. The fineness of CFA did not affect the plastic viscosity h of the mortars. The last statement may be a bit surprising, because it seems that with the decreasing ash fineness, the plastic viscosity h should be also decreasing. However, such an effect was not found—in the investigated area, the fineness of raw or processed CFA did not affect the viscosity. This effect requires further research. It seems that due to the complexity of the CFA system, we are confronted with complex interactions involving the influence of various factors. Yet, on the basis of the conducted research it cannot be confirmed.

**Figure 6:**
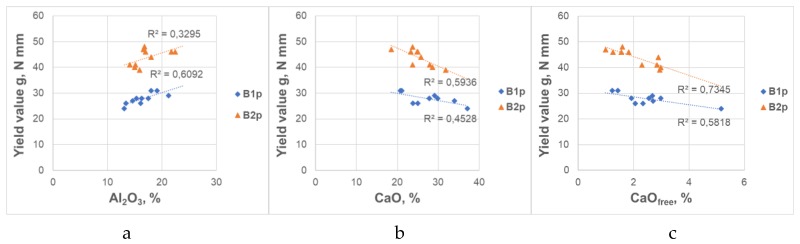
Influence of chemical composition of CFA on the yield value g of mortars. (**a**) Al_2_O_3_, content in CFA, (**b**) CaO content in CFA, (**c**) CaO_free_ content in CFA.

The chemical composition of CFA showed significant variations. Due to the significant impact of the source of CFA on the rheological parameters of mortars, the analysis of the impact of the CFA composition was carried out separately for CFA B1 and B2. Due to the fact that the CFA fineness significantly affected the rheological properties of mortar, the analysis was carried out for ground CFA. The matrix of determination coefficients R^2^ for the relationships between rheological parameters of CFA mortars and chemical composition of CFA is presented in Table 9, and selected relationships in Figure 6. On this basis, it can be concluded that the influence of chemical composition of CFA on the rheology is generally insignificant. Stronger trends can be observed for the influence of Al_2_O_3_, CaO, CaO_free_ content on the yield value g. The increase of Al_2_O_3_ content and the decrease of CaO (and CaO_free_) content in the CFA caused the yield value g of CFA mortars to increase.

## 4. Conclusions

The analysis of the results shows a significant influence of the addition of CFA on the rheological properties of mortars and their variability. The said impact depends on the following factors: the CFA source (different coal combustion conditions, manifested as the differences in the loss on ignition (LoI) and bulk density of CFA) and CFA processing. Ashes from different sources vary due to the differences in combustion processes of coal in boilers, and ashes from different boilers are stored in the retention reservoir (therefore the sample is averaged). The authors do not have information about the combustion process parameters and their impact on ash properties. Since this information is not made available by the power plant, it is not possible to determine the actual process parameters at the time of ash sampling.

The chemical composition of CFA, despite its high variability, was of secondary importance in terms of rheological properties of mortars and their variability. The source and processing of CFA had a significant influence on the yield value g of mortars; their impact on mortar’s plastic viscosity h was negligible. The processing of CFA should be considered as a condition to use it as an additive to concrete.

The coefficient of variation (CV) of rheological properties with the addition of CFA was on the level of 25%. Therefore, it can be concluded that the negative influence of raw CFA on mortar workability combined with high CV of rheological properties with the addition of CFA makes it doubtful to use raw CFA in concrete technology. However, high CV of rheological properties with the addition of CFA can be reduced if appropriate actions are taken such as CFA processing and the control of their source. With respect to the use of processed CFA from a definite source, the CV of rheological properties with the addition of CFA is on the level of 10%, which should be considered as acceptable for practical reasons. The acceptable effect of CFA T (collected from a retention tank) on rheological properties of mortars and its variability is noteworthy. We believe that, despite the differences between CFA from different sources, as a result of mixing in a retention tank, CFA with stable properties can be obtained. However, it is still necessary to control each delivered CFA batch in terms of its impact on the rheological properties of the mix. The presented test results should become an impulse for further, thorough research and analyses.

## Figures and Tables

**Table 1:** Variability of composition and properties of raw calcareous fly ash (CFA), AV—average value, M—median, CV—coefficient of variation.

Component/Property		All CFA			Electrostatic of Boiler B1			Electrostatic of Boiler B2			Retention Tank T		
		AV	M	CV	AV	M	CV	AV	M	CV	AV	M	CV
SiO_2_, %		41.9	41.1	13.4	42.0	40.9	15.4	42.6	42.9	12.5	39.9	40.6	12.0
Al_2_O_3_, %		17.4	16.9	15.5	16.5	16.2	15.5	17.3	16.7	16.0	20.0	20.0	5.2
Fe_2_O_3_, %		4.1	4.0	21.4	4.2	4.1	18.2	3.7	3.4	25.6	4.8	4.8	9.8
CaO, %		26.3	25.4	17.2	27.7	28.0	19.1	25.3	24.9	14.2	25.1	24.2	18.7
MgO, %		1.5	1.5	12.3	1.6	1.5	12.3	1.5	1.6	11.3	1.6	1.6	16.1
SO_3_, %		3.0	2.8	28.2	2.9	2.8	12.7	3.0	2.8	38.8	3.3	3.2	28.4
K_2_O, %		0.19	0.15	67.8	0.26	0.20	69.0	0.13	0.13	13.7	0.16	0.17	26.5
Na_2_O, %		0.24	0.23	26.7	0.25	0.24	21.7	0.24	0.25	28.2	0.21	0.19	40.1
TiO_2_, %		1.37	1.37	14.4	1.39	1.37	14.4	1.39	1.37	14.1	1.28	1.29	17.1
CaO_free_, %		2.23	2.19	43.2	2.51	2.45	43.3	2.12	2.07	36.1	1.79	1.32	62.1
LOI, %		3.4	3.0	33.0	2.8	2.7	6.2	4.5	4.5	20.3	2.3	2.4	18.4
Fineness, % as retention on 0.045 mm sieve	raw	49.2	49.6	15.3	47.5	49.4	11.4	51.0	50.2	17.4	48.9	51.0	19.7
	ground	7.6	5.3	17.9	5.1	4.8	17.6	5.0	5.2	20.2	20.1	20.4	13.0
Water demand %	raw	114	114	5.5	109	110	3.3	120	118	2.9	113	114	2.7
	ground	103	104	2.8	102	103	2.8	105	106	2.4	103	102	1.0
Bulk density kg/m^3^		890	934	14.6	974	978	5.3	757	755	5.8	999	999	7.0

**Table 2:** ANOVA involving the influence of the source of CFA (boilers (B1, B2) and retention tank (T)) on its composition.

Statistics	LoI	SiO_2_	Al_2_O_3_	Fe_2_O_3_	CaO	MgO	SO_3_	K_2_O	Na_2_O	TiO_2_	CaO_free_
*F*-ratio	26.41	0.315	2.895	1.773	0.763	0.418	0.313	2.998	0.648	0.537	0.912
Significance level	0.000	0.734	0.078	0.201	0.479	0.664	0.735	0.072	0.533	0.592	0.417

**Table 3:** ANOVA involving the influence of the source of CFA (B1, B2, T) on its properties.

Statistics	Fineness of CFA		Water Demand of CFA		Bulk Density of Raw CFA
	Raw CFA	Processed CFA	Raw CFA	Processed CFA	
*F*-ratio	0.849	216.4	27.59	4.374	67.31
Significance level	0.442	0.000	0.000	0.026	0.000

**Table 4:** Cement properties.

Cement	SiO_2_	Al_2_O_3_	Fe_2_O_3_	CaO	MgO	SO_3_	K_2_O	Na_2_O	Density, (g/cm^3^)	Specific surface, (cm^2^/g)
CEM I	19.5	4.9	2.9	63.3	1.29	2.76	0.90	0.14	3.1	3500

**Table 5:** Composition of mortars.

Mortar Constituent	Content, g/batch
Cement	450/360
Calcareous fly ash (CFA)	-/90
Water	247.5
Sand acc. EN 196-1 [22]	1350
w/(c + CFA)	0.55

**Table 6:** Rheological properties of reference mortar REF, AV—average value, M—median, CV—coefficient of variation.

Statistics	Yield Value g, N·mm					Plastic Viscosity h, N·mm·s			
	g_5_	g_30_	g_60_	g_90_	g_90_ − g_5_	h_5_	h_30_	h_60_	h_90_
AV	27	34	39	49	22	10.3	10.2	9.0	8.2
M	1.2	1.5	1.7	2.5	1.4	10.3	10.2	9.0	8.2
CV,%	4.3	4.5	4.3	5.2	6.5	4.9	4.4	2.8	3.1

**Table 7:** Variability of rheological properties of CFA mortars. AV—average value, M—median, CV—coefficient of variation, %.

Yield Value g, N·mm Plastic Viscosity h, N·mm·s	All CFA			Electrostatic of Boiler B1			Electrostatic of Boiler B2			Retention Tank T		
	AV	M	CV	AV	M	CV	AV	M	CV	AV	M	CV
**Raw CFA**												
g_5_	54	49	24.9	47	46	8.4	77	78	7.0	49	52	14.4
g_30_	62	62	9.1	62	61	9.9				62	63	8.3
g_60_	73	70	10.7	72	70	10.0				78	76	12.4
g_90_	81	81	4.8	81	81	5.5						
Δg = g_90_ − g_5_	39	39	7.2	38	38	6.4						
h_5_	16.0	16.2	7.5	16.0	16.2	8.3	16.6	16.8	2.8	15.5	15.9	8.5
h_30_	15.7	15.5	8.5	15.4	15.4	7.3				16.5	16.1	10.4
h_60_	15.4	15.8	9.6	15.1	15.7	9.1				16.0	15.8	11.6
h_90_	15.3	15.1	12.1	15.1	14.4	13.5						
**Processed CFA**												
g_5_	35	32	22.6	28	28	6.4	44	45	7.4	31	32	6.5
g_30_	49	48	21.5	39	40	8.6	60	60	7.5	47	48	10.0
g_60_	61	62	18.1	50	50	8.9	71	72	8.0	61	62	6.3
g_90_	73	70	14.9	63	63	4.9	85	86	5.6	69	69	4.8
Δg = g_90_ − g_5_	38	37	10.5	35	36	6.0	41	41	10.3	38	38	5.9
h_5_	15.5	15.3	5.8	15.2	15.1	5.3	15.5	15.4	5.3	16.1	16.3	7.2
h_30_	14.8	14.9	6.4	14.7	14.9	5.2	14.6	14.3	6.8	15.5	15.5	7.2
h_60_	15.1	15.2	7.0	14.6	14.4	3.7	15.3	15.9	8.9	15.9	15.9	4.0
h_90_	14.9	14.7	7.4	14.5	14.5	4.7	15.0	14.4	9.7	15.6	15.6	3.5

**Table 8:** ANOVA for the influence of the source of CFA (B1, B2, T) on rheological properties of mortars.

Statistics	Yield Value g, N·mm					Plastic Viscosity h, N·mm·s			
	g_5_	g_30_	g_60_	g_90_	g_90_ − g_5_	h_5_	h_30_	h_60_	h_90_
**Raw CFA**									
*F*-ratio	37.52	-	-	-	-	0.790	-	-	-
Significance level	0.000	-	-	-	-	0.472	-	-	-
**Processed CFA**									
*F*-ratio	94.22	66.86	43.12	55.75	7.179	1.602	1.289	2.082	1.723
Significance level	0.000	0.000	0.000	0.000	0.004	0.225	0.271	0.083	0.203

**Table 9:** Matrix of coefficients of determination R^2^ for correlations between rheological parameters of CFA mortars and chemical composition of CFA.

CFA Source	SiO_2_	Al_2_O_3_	Fe_2_O_3_	CaO	MgO	SO_3_	K_2_O	Na_2_O	TiO_2_	CaO_free_
**Yield Value g after 5 min**										
B1	0.008	0.609	0.101	0.453	0.001	0.001	0.422	0.000	0.201	0.582
B2	0.027	0.330	0.165	0.594	0.492	0.056	0.528	0.303	0.042	0.735
**Yield Value g increase in 90 min**										
B1	0.004	0.037	0.066	0.091	0.007	0.567	0.007	0.059	0.079	0.079
B2	0.018	0.009	0.078	0.006	0.060	0.007	0.002	0.120	0.036	0.008
**Plastic Viscosity h after 5 min**										
B1	0.000	0.038	0.027	0.028	0.077	0.008	0.158	0.474	0.085	0.035
B2	0.161	0.022	0.064	0.001	0.012	0.107	0.000	0.071	0.097	0.037
**Plastic Viscosity h increase in 90 min**										
B1	0.151	0.011	0.012	0.157	0.243	0.163	0.258	0.000	0.000	0.285
B2	0.025	0.191	0.203	0.289	0.294	0.174	0.141	0.297	0.001	0.142
